# Primary endometrioid adenocarcinoma of the cervix with widespread squamous metaplasia – a potential diagnostic pitfall

**DOI:** 10.1186/1746-1596-2-40

**Published:** 2007-10-25

**Authors:** Lynn Hirschowitz, Chandan Sen, John Murdoch

**Affiliations:** 1 Department of Cellular Pathology, Southmead Hospital, Bristol, BS10 5NB, UK; 2 Department of Gynaecological Oncology, St Michael's Hospital, Southwell Street, Bristol BS2 8EG, UK

## Abstract

**Background:**

Uterine or endocervical biopsies that contain endometrioid adenocarcinoma with widespread squamous metaplasia are usually of endometrial origin. The presence of squamous metaplasia is said to be helpful in distinguishing endocervical from endometrial adenocarcinomas in small biopsy samples.

**Case presentation:**

A 51-year-old woman presented with post-coital and post-menopausal bleeding. Biopsy of a friable lesion in the proximal endocervical canal revealed an endocervical adenocarcinoma of endometrioid type with widespread squamous metaplasia. The latter feature initially raised the possible diagnosis of a primary endometrial adenocarcinoma. However, immunohistochemical marker studies indicated a diagnosis of primary endocervical adenocarcinoma of endometrioid type and this was confirmed at hysterectomy.

**Conclusion:**

Squamous differentiation is not well documented in endocervical adenocarcinomas of endometrioid type and, when widespread, may represent a diagnostic pitfall for pathologists. Interpretation of small biopsies from the endocervical canal on the basis of morphology alone may lead to misdiagnosis and inappropriate surgical management.

## Background

Differentiating between endocervical and endometrial adenocarcinoma in small pre-operative endocervical and endometrial specimens may be difficult: endometrial adenocarcinomas may involve the endocervix and endocervical neoplasms may be sampled en-route to the endometrial cavity, and primary tumours at both of these sites may show histological similarities. Endometrioid adenocarcinomas are a recognised subtype of tumours of the cervix [1] and mucinous differentiation may be seen in both endocervical and endometrial adenocarcinomas; the presence of mucin is not therefore a discriminating feature. In contrast, squamous differentiation is a well-recognised feature of endometrial but not endocervical adenocarcinomas. In his review of the pathology of endocervical glandular lesions, McCluggage states that one of the clues to a primary endometrial lesion is the presence of benign squamous cells [2].

We describe a patient from whom friable tissue removed from the endocervical canal showed adenocarcinoma with an endometrioid morphology, focal mucinous differentiation and widespread benign squamous (morular) metaplasia suggestive of a primary endometrial adenocarcinoma but who was subsequently found to have a primary endocervical adenocarcinoma of predominantly endometrioid type. Squamous differentiation in primary endocervical adenocarcinomas of endometrioid type is poorly documented but can be a prominent feature.

## Case presentation

A 51-year-old woman presented with post-coital and post-menopausal bleeding. Gynaecological examination revealed a friable lesion in the endocervical canal. Clinically a diagnosis of an endocervical neoplasm was raised. The lesion was biopsied and two friable, haemorrhagic tissue fragments up to 10 mm in diameter were submitted for histology.

## Results

**Figure 1:**
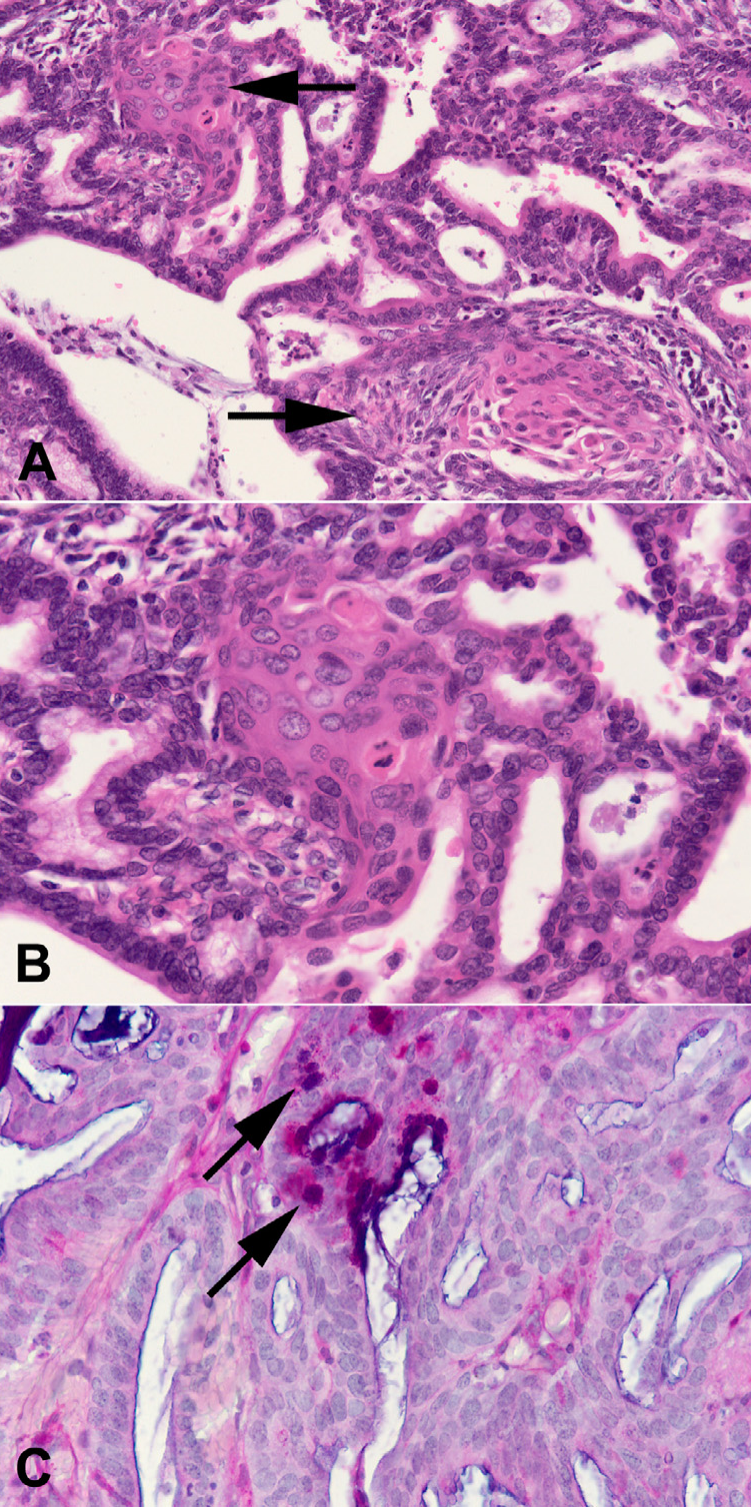
(**A**) and (**B**) Endometrioid adenocarcinoma with a cribriform glandular architecture. Foci of benign squamous (morular) metaplasia (arrows) are present between glandular structures. Haematoxylin and eosin; objective magnification ×20 (A), ×40 (B). (**C**) Alcian blue and PAS-positive cytoplasmic mucin (arrows) in the mucinous but not the endometrioid glands. Objective magnification ×40.

Histology revealed adenocarcinoma of endometrioid type with a complex tubulopapillary and cribriform architecture. The tumour showed focal mucinous and more widespread benign squamous differentiation (morular metaplasia) (Fig. 1a, b). The combined FIGO grade was I. Alcian blue- and PAS-positive cytoplasmic mucin was present in the mucinous but not the endometrioid areas (Fig. 1c). The biopsy did not include any normal endocervical or endometrial tissue, any endocervical fragments with CGIN, or hyperplastic endometrial tissue. Since endometrioid endometrial adenocarcinomas may show mucinous differentiation and squamous metaplasia, the possibility of a primary endometrial adenocarcinoma was considered on the basis of the histology. However, as an endocervical neoplasm had been considered clinically, immunohistochemical marker studies were performed to exclude the possibility of a primary endocervical neoplasm.

Sections were immunostained using a standard strepavidin-biotin technique, with antibodies to carcinoembryonic antigen (CEA; polyclonal, Dako, Ely, UK, 1:10,000 dilution), p16 (Novocastra, Newcastle-upon-Tyne, 1:100), oestrogen receptor (Vector Laboratories, Peterborough, UK 1:100) and vimentin (Dako, UK, 1:600). Both the squamous morules and the mucinous areas marked strongly for CEA. There was much less labelling of the endometrioid areas, within which staining was largely confined to the luminal surface of some of the glands. Only very focal cytoplasmic staining was observed for vimentin, approximately 10% of the tumour cell nuclei were weakly immunopositive for oestrogen receptor, and there was strong cytoplasmic and nuclear labelling of both the squamous and glandular elements for p16. Despite the histological features that had suggested primary endometrial adenocarcinoma (with mucinous differentiation and widespread squamous metaplasia), the immunohistochemical profile favoured a primary endocervical neoplasm.

Radiological investigations indicated that the carcinoma was located high in the endocervix and a radical hysterectomy was performed. The resected specimen was received at a nearby hospital and not photographed. However, it was extensively sampled for histology. All of the sections were reviewed by the first two authors and a diagnosis of a FIGO stage IB1, grade I adenocarcinoma of mixed endometrioid and mucinous subtype was identified on the posterior cervical lip, with associated lymphovascular invasion. Multiple foci of squamous metaplasia were present in the adenocarcinoma in the areas with an endometrioid morphology. The histological appearances were identical to those in the original biopsy. There was no adjacent CGIN or CIN in the cervix, and no evidence of a primary endometrial neoplasm. The paracervical and parametrial tissues and all of the resected pelvic lymph nodes were tumour-free.

## Discussion

The distinction between primary endometrial and endocervical adenocarcinomas is important for optimal patient management. Localized endocervical adenocarcinoma is best treated by chemoradiation or radical hysterectomy and pelvic lymphadenectomy. The treatment of endometrial adenocarcinoma depends on the stage of disease and may not involve such extensive surgery. It can be difficult to differentiate between primary endometrial and endocervical adenocarcinomas in small endometrial or endocervical samples. Squamous differentiation, most commonly in the form of benign squamous metaplasia, is a well-recognised feature of endometrial adenocarcinomas of both primary endometrial and ovarian origin. The present case illustrates that widespread squamous differentiation may also occur in primary endometrioid adenocarcinoma of the cervix.

Endometrioid adenocarcinomas of the cervix are a subtype of primary endocervical adenocarcinoma [1]. Although Young and Clement [1] regard this subtype of endocervical adenocarcinoma to be uncommon, others have reported it to account for up to 30% of primary endocervical adenocarcinomas [3]. Alfsen et al [4] reported an increase in the proportion of non-squamous carcinomas of the cervix over the past few decades in Norway, endometrioid adenocarcinoma accounting for 21%.

Endometrioid endometrial adenocarcinomas can show both squamous and mucinous differentiation. Zaino [5] reported squamous differentiation in up to 25% of endometrioid adenocarcinomas but others [3] describe this in 20–50% or more. Mucinous differentiation is also described in primary endometrial adenocarcinomas [6-8]. Because of the endometrioid morphology and squamous and mucinous differentiation, the histology of the endocervical biopsy was thought initially to favour primary endometrial adenocarcinoma.

The immunohistochemical differentiation of endocervical from endometrial adenocarcinoma has been described in several publications [2,9-11] and a limited marker panel has been recommended. More extensive immunohistochemical investigations are occasionally warranted for rarer endometrial and cervical tumours [12,13]. In the present case, despite the morphology, immunohistochemical marker studies favoured a diagnosis of primary endocervical adenocarcinoma – a diagnosis confirmed at hysterectomy. This case emphasizes the value of immunohistochemical marker studies, particularly when the clinical details and histological findings seem discordant; endometrioid endocervical adenocarcinoma should be considered even in the presence of squamous metaplasia. Squamous metaplasia may be a prominent feature of endometrioid tumours, whatever their primary anatomical site of origin.

## Competing interests

The author(s) declare that they have no competing interests.

## Authors' contributions

LH drafted the manuscript. CS reviewed the histology and helped to draft the manuscript. JM contributed to the clinical background.

All authors read and approved the final manuscript.
